# Influence of Adhesive Strength, Fatigue Strength and Contact Mechanics on the Drilling Performance of Diamond Coating

**DOI:** 10.3390/ma13061402

**Published:** 2020-03-19

**Authors:** Naichao Chen, Musen Liu, Ping He

**Affiliations:** 1 School Energy and Mechanical Engineering, Shanghai University of Electric Power, Shanghai 200090, China; sdlwlms@mail.shiep.edu.cn (M.L.); heping@shiep.edu.cn (P.H.); 2 Shanghai Key Laboratory of Materials Protection and Advanced Materials in Electric Power, Shanghai 200090, China

**Keywords:** adhesive strength, fatigue strength, contact mechanics, diamond coating, lifetime

## Abstract

Adhesive strength of the coating significantly affects the lifetime of the coating. However, it is still inevitable for the coating, even with strong adhesive strength, to peel off from the substrate after working for a while. In this work, fatigue and wear behaviors were employed to analyze the effect on the mechanics of coating and contribute to a fundamental understanding of peeling of the coating. A small-size Co-cemented tungsten carbide drill bit was selected as the examined substrate to fabricate the diamond coating. Roughening pretreatment with a diamond slurry combined with ultrasonic vibration was performed for the substrate surface to enhance adhesive strength. Meanwhile, a diamond coating without roughening pretreatment was also fabricated for comparison. The lifetime and quality of the coating were evaluated by the drilling test. Although the diamond coating could grow on the substrates with and without roughening pretreatment, the diamond coating with roughening pretreatment possessed a higher lifetime and stronger wear resistance than that without roughening pretreatment. We found that both substrates with and without roughening pretreatment exhibited a coarse surface, whereas the roughening pretreatment could remove the original machined surface of the substrate and thus make the near surface with numerous integrated crystalline grains become the new topmost surface. This increased the contact area and surface energy of the interface, leading to the improvement of adhesive strength. Finally, fatigue strength and contact mechanics were studied to trace the changes in the stress of the diamond coating in the whole process of drilling from a theoretical point of view. We suggest that fatigue strength and contact mechanics may play vital roles on the durability and peeling of the coating.

## 1. Introduction

To date, diamond is regarded as the hardest material in the world, which has attracted the intensive attention of scholars and engineers to adopt it as a protective coating in industrial fields [1,2,3,4]. Due to the difference in lattice parameters and coefficients of linear expansion between the substrate and coating, the structure of the coating/substrate interface is very complex and the interface shows a relatively weaker strength than the bulk materials, which leads to the most frequent occurrence of the peeling of the coating. It is still a considerable challenge for the diamond coating to avoid peeling because the diamond/substrate adhesive strength is not strong enough to undergo the external load [5,6]. Hence, high adhesive strength has become a vitally technical requirement for the coating prior to practical application [7].

Not all materials can be used as substrates on which to deposit a diamond coating. Only several materials such as Co-cemented tungsten carbide (WC–Co), silicon nitride, silicon carbide, silicon, etc. could easily grow the diamond grains by the chemical vapor deposition (CVD) method [8,9]. For the substrate, pretreatment is recognized as a necessary and important step before fabricating the diamond coating, since the morphology of the substrate significantly affects the diamond nucleation and mechanical properties of the coating including adhesive strength [10]. Compared to WC–Co, the ceramic substrate has a relatively easier pretreatment, which is only to polish the substrate surface by hard paper combined with diamond powders and thereafter to rinse the substrate to clean its surface [9,11]. However, this procedure cannot be suitable for the WC–Co substrate [12] because the Co element in the WC–Co surface will promote graphite to form on the interface [13]. Hence, removing the superficial Co element is of great importance to grow the diamond coating on a WC–Co substrate [14]. According to the current technique, chemical etching is classified as an effective method to eliminate the Co element from the WC–Co surface [15,16]. Meanwhile, it can also roughen the substrate surface, facilitating the enhancement of diamond/WC–Co adhesive strength via mechanical interlock [17].

It is noted that WC–Co is a relatively hard material that easily breaks due to weak flexibility, especially for the thin and small size WC–Co drill bit, which often has detrimental consequences on how to roughen the WC–Co surface. Furthermore, for substrates with complex shapes, it is not easy to roughen the surface by automatic polishing machines. Hence, it is of interest to explore effective pretreatment for micro-sized substrates with complex shapes to deposit a CVD diamond coating [18,19,20]. After the chemical etching, Polini et al. [19] only washed the substrates with distilled water in an ultrasonic bath, while Almeida et al. [20] ultrasonically seeded the drill bits in a suspension of diamond particles in n-hexane for 1 h. However, the effect of pretreatment on the mechanical properties of diamond coating have been less investigated. Additionally, after working for a while, the diamond often peels off from the substrate [21,22], even though the diamond/substrate interface exhibits strong adhesive strength. As far as the experimental results are concerned, it is not surprising that only increasing adhesive strength may not be the most effective method in avoiding the peeling of the coating. In fact, fatigue and wear, which must be mentioned, always exist for diamond-coated tools in the process of industrial applications, which in turn change the morphology of the coating. In this way, the original properties of the coating will deteriorate gradually with the time of fatigue and wear, and thus accelerate the occurrence of the coating peeling. Hence, further efforts should be put to studies on the influence of fatigue and wear on the peeling of the coating.

The purpose of this work was to investigate the effect of adhesive strength, fatigue strength, and contact mechanics on the peeling of the coating. A micro-sized WC-Co drill bit was selected as the substrate because of its complex shape and small size. The pretreatments with and without roughening pretreatment were performed for the substrate before fabricating the CVD diamond coatings. Drilling tests were conducted to evaluate the properties of the diamond coatings. The surface morphologies of the substrate were observed to analyze the diamond/WC–Co adhesive strength. Finally, fatigue strength and contact mechanics were employed to understand the mechanism of the peeling of the coating from a theoretical point of view.

## 2. Experimental

### 2.1. Depositing Chemical Vapor Deposition (CVD) Diamond Coating

In this work, the WC–Co (YG6) micro-sized drill bit with a diameter of 1 mm was chosen as the substrate to deposit the diamond coating because it is widely used to machine the holes in many industrial fields [10,12]. The micro-sized drill bit was pretreated by a two-step chemical etching method to remove the Co components from the surface of the WC–Co substrate. First, the WC–Co drill bits were put in an ultrasonic vessel with Murakami’s reagent (10 g K_3_[Fe(CN)]_6_ + 10 g NaOH + 100 mL H_2_O) for 30 min, and then etched with Caro’s acid (30 mL H_2_SO_4_: 70 mL H_2_O_2_) for 1 min. Murakami’s reagent was used to attack the WC grains and roughen the substrate surface together. Caro’s acid oxidizes the binder to soluble Co^2+^ compounds, thus decreasing the surface cobalt content [23].

It has been noted that roughening the substrate is an important pretreatment to deposit the CVD diamond coating. As for the small diameter and complex shape, we adopted two approaches to deal with the etched drill bits in order to avoid destruction and fracture of the drill bit. One is to keep its original status without any roughening treatment; the other is to immerse drill bits into a diamond slurry in an ultrasonic vessel for 20 min, which imitates the polishing processing, although the polishing force loaded on the surface of a drill bit is relatively small.

The diamond deposition was performed by the conventional hot filaments CVD (HFCVD) technique in an in-house bias-enhance reactor (Suzhou Jiaozhuan Ltd. Co., Suzhou, China). Six tantalum wires were used as the hot filaments to offer the high temperature for CVD chemical reactors to grow diamond. The cutting bars of the drill bits were inserted in the holes of the fixture placed on the clockwise rotating platform. All the drill bits and the fixture were put below the hot filaments, and the distance between the hot filaments and the nose of the drill bit was fixed at about 12 mm. All the wires were dragged by the spring to maintain the straight status and keep a constant distance to the substrate at high temperature. During the deposition, the substrate sustains 800–900 °C, which provides the suitable temperature for growing the diamond grains.

An acetone–H_2_ gas mixture was used as the reaction gas to deposit the diamond coating. Here, the partial H_2_ gas acts as the carrier gas to flow into the volatile liquid acetone in order to bring the vapor acetone into the CVD reactor chamber, and the remaining H_2_ gas was used as the active gas to motivate the carbon source (acetone) to generate the diamond grain. Microcrystalline diamond (MCD) coatings were grown by assigning the deposition parameters, as shown in Table 1. The low pressure of 1.5–2.0 kPa and bias current of 4.0 A were adopted in the nucleation period to improve the diamond nucleation density. The concentrations of carrier gas and active gas were set as 80 and 200 sccm, respectively. Subsequently, in the growth period, the bias current and the gas pressure were changed manually to 3.0 A and 4.0–5.0 kPa, respectively. This provides a suitable condition to grow the diamond grains. Meanwhile, the carrier gas also increased by 10 sccm to enhance the nucleation ratio of the diamond grains. The growth time was set as 4 h.

The surfaces morphologies of the as-deposited diamond coating were characterized by SEM (Hitachi Ltd., Tokyo, Japan). In order to study the adhesive strength, the surface and cross-sectional morphologies of the substrate with and without roughening pretreatment were also observed by SEM. Due to the small diameter and brittleness, we manually broke the drilling bit along the radial direction to measure the cross-sections. Raman analysis was conducted to evaluate the purity of the diamond and graphite phases in the coating by Horiba Jobin Yvon XploRA Raman spectroscopy (Horiba Jobin Yvon Ltd., Paris, France) with a spectral resolution of 1.2 cm^−1^ using an Ar^+^ laser with an excitation wavelength of 632.8 nm, which has also been used in previous literatures [12].

### 2.2. Machining Test

In this work, printed circuit board (PCB) with a thickness of 2 mm was selected as the machining material to test the quality of the coating [16], because this type of micro-sized drill bit is often used to drill the PCB holes. During drilling of the PCB holes, the diamond/WC–Co interface undergoes the cutting force again and again, which is a convenient and straightforward way to test the strength of the interface.

Machining tests were performed on a carving turning machine (Shanghai Peidi Precision Machinery Co. Ltd., Shanghai, China) with a 24,000 rpm spindle speed, which is a high performing multi-operation CNC lathe with three controlled axes. The drill bits are grasped by a three-jaw chuck. The cutting parameters were set as follows: spindle speed of 24,000 rpm and feed rate of 100 mm/min. The mechanical properties of the diamond coating were evaluated by an analysis of tool wear and durability. The criteria used for the tool wear was to measure the maximum width of flank wear width because the tool is not regularly worn and chipping is the main type of failure [24]. All the flank wear widths were measured after each machined 200 holes by tool microscopy with a digital image processing system. The machining processes were terminated once the diamond coating peeled off from the substrate.

## 3. Results and Discussion

### 3.1. Characterization of Diamond Coating

**Figure 1:**
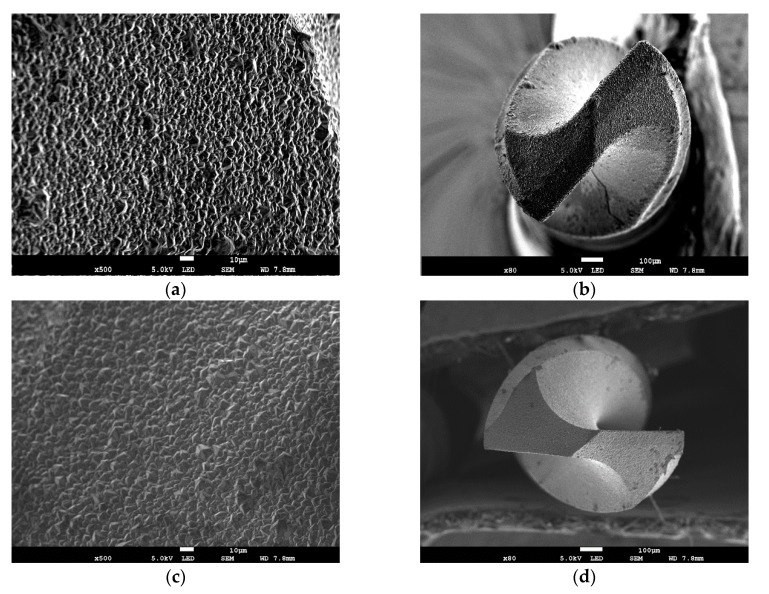
Scanning electron microscopy (SEM) images of surface morphology and whole shape of the (**a**,**b**) microcrystalline diamond (MCD) and (**c**,**d**) roughening pretreatment microcrystalline diamond (RMCD) coated drill bits, respectively.

Figure 1 shows the surface morphologies and whole shapes of the MCD-coated drill bits with and without roughening pretreatment. In order to conveniently elucidate the following context, the MCD coating with roughening pretreatment is called the RMCD coating. It can be seen that the well-faced diamond crystallites with grain size of 3–5 μm cover on the surfaces of the drill bits with and without roughening pretreatment, suggesting that the roughening pretreatment does not affect the growth of the diamond coating. Both diamond coatings displayed the typical features of MCD coating with micro-sized diamond crystalline grains.

**Figure 2:**
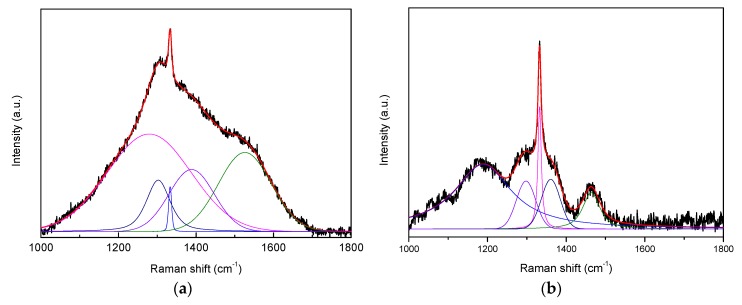
Raman spectra of the (**a**) MCD and (**b**) RMCD coating.

Raman analysis was conducted to evaluate the material structures of the as-deposited diamond coating. Figure 2 shows the Raman curves obtained from the MCD coating and the RMCD coating. An optimized fitting procedure of the spectra includes five bands (1260, 1302, 1333, 1384, and 1536 cm^−1^) in Figure 2a, and five bands (1186, 1295, 1332, 1359, and 1463 cm^−1^) in Figure 2b. Both coatings involved a narrow and sharp peak at ~1332 cm^−1^, which was assigned to the natural diamond Raman line [25]. The MCD coating showed less change in the diamond Raman line and a lower Raman peak than the RMCD coating, implying that the diamond quality of the RMCD coating was slightly better than that of the MCD coating. Roughening pretreatment seemed to exhibit the position effect on the quality of the diamond. However, some sp^2^-phases [25] also co-existed inside both coatings, and these Raman peaks were assigned to trans-polacetylene (1186 cm^−1^) [26,27], with the maximum in the phonon density of states (PDOS) of diamond or trans-polyacetylene (1260, 1295, and 1302 cm^−1^) [27,28], the D peak from amorphous carbon (1359 cm^−1^) [29], trans-polyacetylene (1463 cm^−1^) [26,27], and the G peak of amorphous carbon (1536 cm^−1^) [29]. This serves as a possible indicator of structural changes in the diamond coating, which led to the difference in the mechanical properties of the coating. Here, we do not further discuss the reason for the formation of the sp^2^-phases in this work, and only focused on the peeling properties of the coating.

### 3.2. Drilling Performances of the CVD Diamond Coated Micro Drill Bit

**Figure 3:**
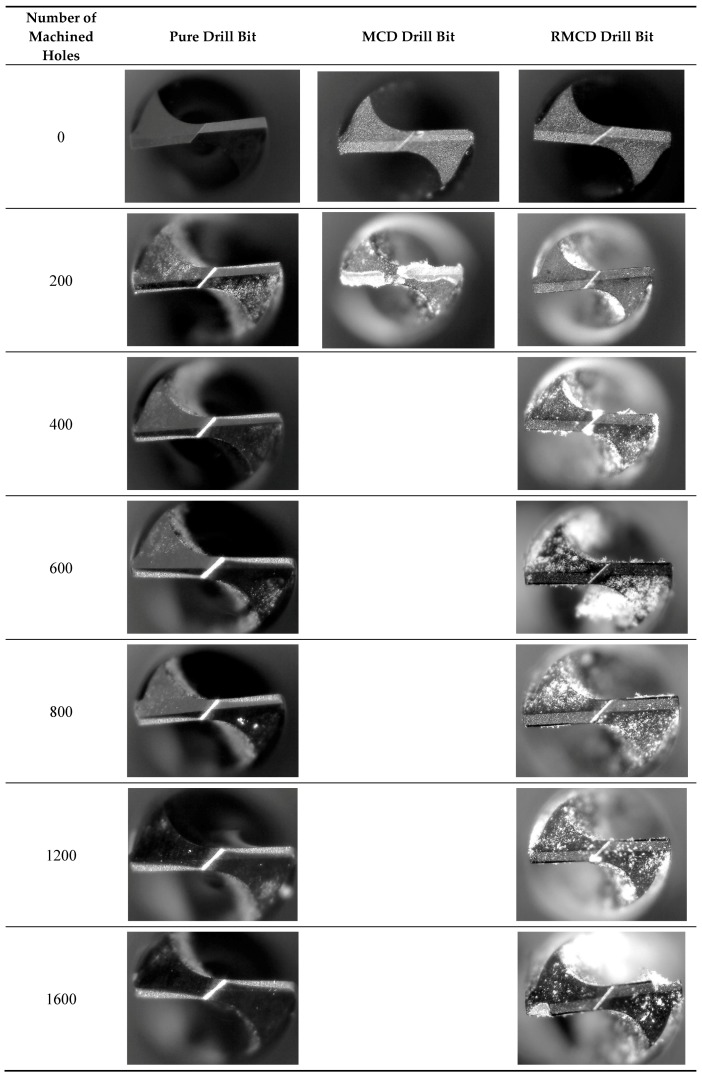
Shapes of the pure, MCD, and RMCD drill bits after machining the printed circuit board (PCB) material.

**Figure 4:**
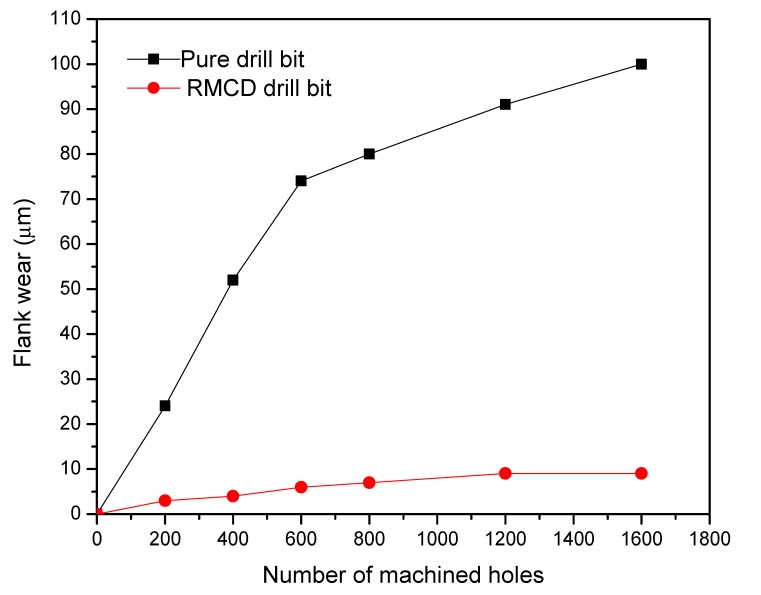
Flank wear width as a function of the number of machined holes by the pure and RMCD drill bits.

In this work, the pure WC–Co drill bit without a diamond coating was also examined for comparison. Figure 3 plots the shapes of the pure, MCD, and RMCD drill bits after each machining 200 holes. Some machined chips adhere on the drill bit. Figure 4 shows the flank wear widths of pure and RMCD drill bits as a function of the number of machined holes. The MCD drill bit is not shown here because the coating peeled off from the substrate after only machining 200 holes. It can be clearly observed that the flank line of the pure drill bit was generally worn and torn with the increase in the number of machined holes. The flank wear width rapidly increased to 75 μm after machining 600 holes and slowly reached 100 μm after machining 1600 holes. As shown in Figure 4, the RMCD drill bit presented remarkably stronger wear resistance than the pure drill bit. Although the drill bit without the roughening pretreatment could fabricate the MCD coating, the adhesive strength was proven to be considerably weak. That is to say, this coating was an unsuccessful product as cutting tool, which cannot satisfy the realistic requirement. While the RMCD coating did not have the same machining performance, the long lifetime and low flank wear width obviously indicated its perfect mechanical properties. After machining 1600 holes, the flank wear width was less than 10 μm. Under this condition, the pure drill bit had a flank wear width of about 100 μm. The RMCD coating improved the wear resistance of about 10 times for the pure drill bits. Unfortunately, the RMCD coating could not avoid peeling after machining 1600 holes.

**Figure 5:**
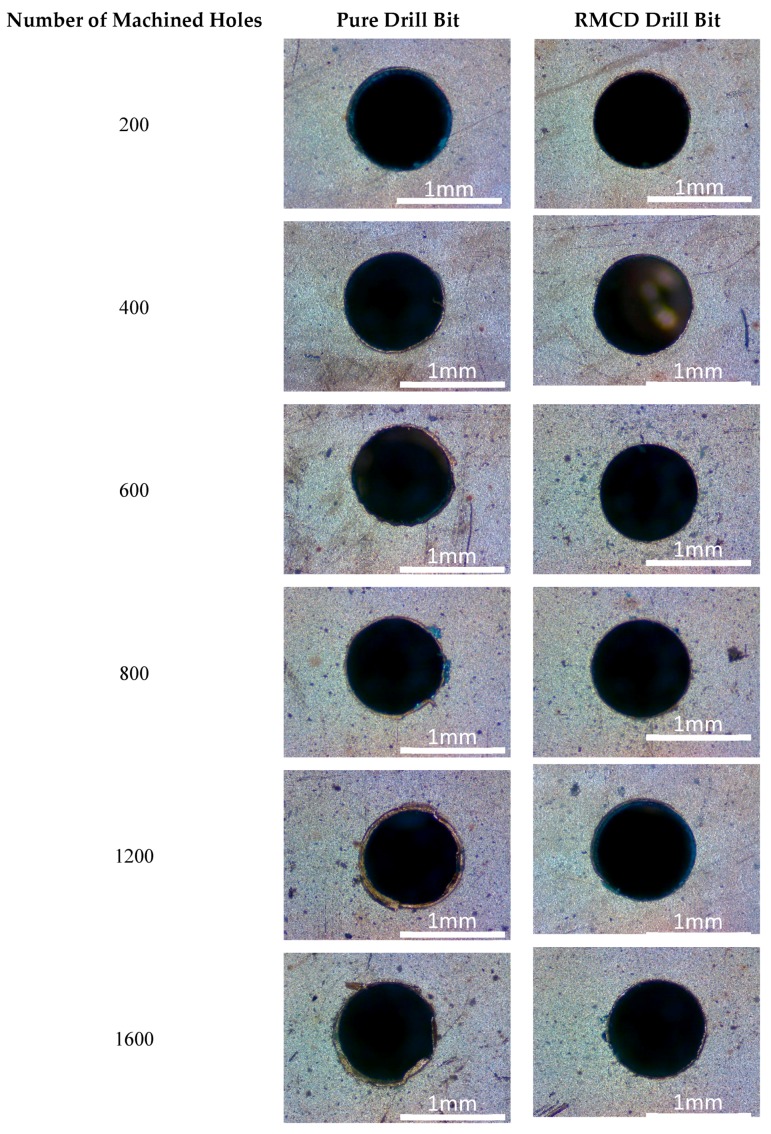
Shapes of the machined holes by the pure and RMCD drill bits.

Figure 5 shows the shapes of the machined holes after each machining 200 holes by the pure and RMCD drill bits, respectively. For the pure drill bits, after machining the initial 200 holes, the shape of the hole was not an absolute circle, and some small burrs were found in the inner surface of the hole, indicating that the pure drill bit was not very sharp when drilling the PCB materials. The wear changed the shape of the drill bit and thus blunted the pure drill bit. As the number of machined holes increased, the pure drill bit became blunter and blunter. After machining 600 holes, a hole occurred at the wrinkle edge; and some large burrs connected with the hole edge after machining 1600 holes. For the RMCD drill bit, the machined holes had the less burrs than those using the pure drill bit. Burrs were not found in the inner surface of the hole after machining 800 holes, suggesting that the integrated-coated drill bit with low flank wear width is a very important factor in obtaining the perfect machined hole. After machining 1200 holes, the hole contour had a small change and did not show a relatively circular shape, which may have resulted from the wear of the drill bit. In this way, after continuing to machine 400 holes, some burrs were present in the inner surface of the hole as the peeling of the coating took place. It can be seen that peeling is still a main detrimental barrier for the quality of the machined hole.

### 3.3. Adhesive Strength and Surface Morphology of Substrate

**Figure 6:**
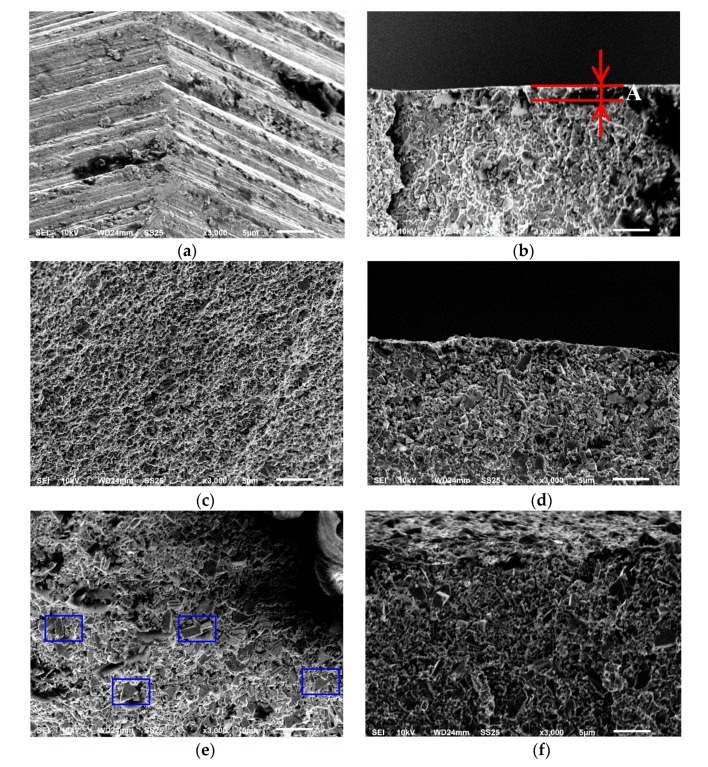
SEM images of the (**a**) surface and (**b**) cross-sectional surface of the original substrate, (**c**) surface and (**d**) cross-sectional surface of the substrate after chemical etching pretreatment, (**e**) surface and (**f**) cross-sectional surface of substrate after chemical etching and roughening. The red lines denote the thickness of the machined layer A made by the mechanical treatment. The blue rectangle points out the large-sized crystalline grains.

The experimental results demonstrate that the roughening pretreatment is very important in enhancing the mechanical properties of the coating. In order to understand the mechanism of such improvement, the surface morphologies of the substrate before and after pretreatment were compared to investigate the adhesive strength, as shown in Figure 6. For the pure drill bit, there was a very rough surface that is determined by the surface treatment of the vendor, and the processing texture can be clearly observed. The cross-sectional image shows that the pure drill bit had a machined layer (A) with a ~1 μm thickness made by the mechanical treatment. After the two-step chemical etching pretreatment, the surface topography of the drill bit showed a manifest change. The processing texture was eliminated by the chemical etching pretreatment, and the surface became very coarse and turned toward the hill-valley morphology. The cross-sectional surface indicates that the depth of the valley was slightly small. The A layer was only etched partially, because the almost linear surface profile along the cross-sectional direction implied the existence of a machined surface. The near-surface and subsurface still maintained their original status. For the substrate by roughening pretreatment, the surface appeared to have some large-sized crystalline grains (see the blue rectangle in Figure 6e). As a result, the surface roughness increased remarkably due to the formation of a new surface. The cross-sectional image shows that the A layer disappeared as the roughening pretreatment punctured the surface and thus exposed the near-surface as the new surface. Here, it is worth noting that the etching treatment aims to reduce the deleterious effect of Co on diamond coating adhesion. The thickness of the etched zone is important, because the remaining porosity in the cemented carbide results in reduced layer adhesion. In this work, roughening treatment did not affect the diamond fabrication because the RMCD coating could grow on the substrate, and the drilling performance was also good. The effect of Co seemed to be very weak on diamond coating adhesion. Hence, it is reasonable to assume that the roughening pretreatment slightly affected the produced etching zone and the superficial Co content.

It was found that both substrates with and without roughening pretreatment had a coarse surface. Our experimental results showed that the diamond coating could grow on these two substrates, and the as-deposited diamond coatings had similar surface morphologies. However, the great discrepancy in cutting performance of the MCD and RMCD drill bits implied that only the coarse surface of the substrate does not seem to ensure the quality of the diamond coating. Perhaps the detailed surface morphology of the substrate should be considered in the design process of increasing the adhesive strength of the coating. In this work, the new surface derived from the near-surface consisted of many integrated crystalline grains, rather than the machined surface, that drastically increased the grain boundaries and contact area, which is greatly beneficial to improving the adhesive strength of the coating. Meanwhile, such grains also increase the surface energy [30] of the substrate, which is tightly associated with the chemical bond between the diamond and substrate. In summary, the roughening pretreatment can offer a large contact area and high surface energy for the substrate, by which the diamond/WC–Co adhesive strength is enforced. Meanwhile, only the coarse surface was not sufficient for the substrate to fabricate the high quality of the coating, so we should also focus on the detailed surface morphology of the substrate.

### 3.4. Fatigue Strength and Contact Mechanics of Diamond Coating

In this work, the effects of fatigue and wear on the peeling of the coating were investigated by an analysis of the fatigue strength and contact mechanics from a theoretical point of view. As above-mentioned, the surface morphology of the substrate is tightly associated with the adhesive strength of the coating, which affects the lifetime of the drill bit. As shown in Figure 3, after machining 1200 holes, the RMCD drill bit exhibited an integrated coating (no peeling) only with a small flank wear width (<10 μm), suggesting that it can be suitable to machine PCB without the potential risk of peeling. However, the results prove that this hypothesis is not correct. After machining 1200 holes, the integrated RMCD coating indicates that the adhesive strength of the coating was strong enough to undergo the external load. However, after machining a further 400 holes, the occurrence of peeling suggests that the adhesive strength of the coating does not suffer from the drilling force that always keeps a constant value, implying that one possible reason is the decrease in the adhesive strength of the coating during drilling. Hence, we believe that fatigue strength [31,32], rather than adhesive strength, may be more suitable to evaluate the mechanical behavior of coatings when it drills the holes again and again. Certainly, adhesive strength is also vital in terms of the peeling of the coating, which can be proven by our experimental results of the MCD coating. From the perspective of industrial application, adhesive strength can be regarded as a fundamental parameter, and fatigue strength is of crucial technological importance in the lifetime of the coating, which directly determines the cost and feasibility of the coating. Currently, much effort has been put into studies on enhancing the adhesive strength of the coating. However, if the fatigue strength is not good, the coating, even with high adhesive strength, is still out of use in many engineering applications due to its short lifetime. Therefore, adhesive strength and fatigue strength seem to require the capability of a diamond coating to withstand static and dynamic loads during drilling, respectively.

**Figure 7:**
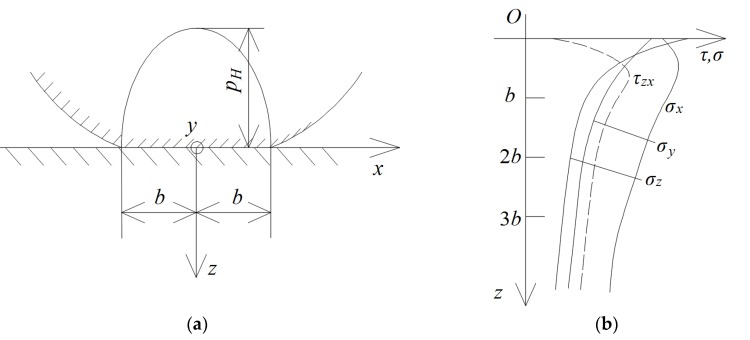
Images of (**a**) Hertz elastic model of two contact counterfaces and (**b**) stress as a function of subsurface position, where *p_H_* is the maximum stress, and *b* is the half width of the contact line.

**Figure 8:**
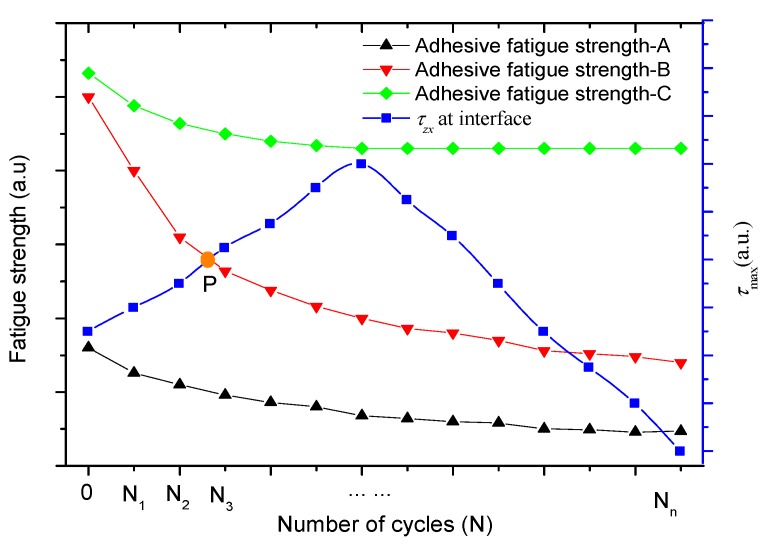
Fatigue strength and shear stress (*τ_zx_*) of the interface as a function of the number of cycles.

According to the principles of the mechanics, the peeling derived from the mismatch between the external force and the adhesive strength of the coating. Fatigue behavior weakened the resistance to external force as the adhesive strength decreased [31]. In this work, the Hertz elastic model and stress of two contact materials [33] (Figure 7) were employed to evaluate the mechanical trend of the coating during wear. Shear stress is often considered as a very deteriorated influence on the contact-fatigue resistance of the interface [32]. The stress trace indicates that the maximum shear stress (*τ*_max_ = 0.301 *p*_H_), located at z = 0.786b, which is in the near surface, rather than the interface or surface, as shown in Figure 7b [33]. In fact, wear existing at the sliding counterfaces constantly destroys the original surface, and thus forms the new surface. Thus, the thickness of the material will gradually decrease once the wear procedure occurs. Compared to the bulk material, the coating is sensitive to its thickness as it is a very thin material. With the decrease in the thickness of the coating, the location of *τ*_max_ will also change from the near surface to the subsurface, according to the Hertz elastic theory. In this way, the shear stress at the interface was not a constant value. Figure 8 shows the shear stress trend as a function of the number of cycles (here, we assumed that the thickness of the coating was larger than 0.786b). Here, all the curves were theoretically postulated to clearly plot the possible and potential relation between fatigue strength and contact mechanics, by which we qualitatively investigated the coupling effect of fatigue and wear on the peeling of the coating. Three types (A, B, and C) of fatigue strength were assumed, and a convex shape was assigned to the shear stress curve due to the occurrence of the transfer of the location of τmax toward the substrate, as shown in Figure 8. The maximum magnitude of fatigue strength A was less than the initial shear stress of the interface (N = 0). The coating will peel immediately off from the substrate, which is in reasonable agreement with our experimental test of the MCD coating, as the coating cannot provide a sufficiently high adhesive strength to withstand the external load. For fatigue strength B, the P intersection between fatigue strength and shear stress can be regarded as the threshold of peeling, which is similar to the behavior of the RMCD coating and the previous experimental observations [21,22]. Under this condition, if the thickness of the coating can keep its initial value without wear, the peeling will be postponed and the lifetime of the coating will be prolonged. Finally, the C fatigue strength can be classified as the best candidate coating in terms of industrial applications. Peeling did not occur during the whole process of the fatigue test. Coating failure mainly comes from wear, until the coating is worn down. As illustrated above, we suggest that fatigue strength and contact mechanics should be emphasized during the discussion on the mechanism of peeling.

## 4. Conclusions

For micro-sized and complex shape substrates, different pretreatments are performed to prolong the lifetime of the as-deposited coating, which is the emphasis in industrial fields. In this work, the WC–Co drill bit with Ø1 mm was selected as the examined sample to fabricate the CVD diamond coating. A roughening pretreatment with diamond slurry in the ultrasonic vessel was proposed to roughen the surface of the substrate. The results showed that a diamond coating can be fabricated on the WC–Co drill bits with and without roughening pretreatment. Machining PCB tests showed that the peeling took place for the MCD coating after machining the initial 200 holes. For the pure drill bit, the flank wear widths rapidly increased with the increase in the number of machining holes. After machining 1600 holes, the flank wear width reached about 100 μm. For the RMCD coating, a very low flank wear width was found in the whole process of drilling, suggesting that such a coating has high adhesive strength and strong wear resistance. However, peeling still occurred after machining 1600 holes. The shapes of the machined holes indicate that the RMCD coating could make smooth and circular holes. Therein, both substrates with and without roughening pretreatment had a coarse surface. However, roughening pretreatment can eliminate the original machined surface and thus expose the near surface as the new topmost surface that consisted of many integrated crystalline grains. Thus, there was a dramatic improvement over the contact area and surface energy of the substrate, by which the quality of the coating is enhanced. Hence, both the surface roughness and surface morphologies should be noted during the deposition of the diamond coating. Finally, fatigue strength and contact mechanics were employed to contribute to a better understanding of the peeling of the coating. Adhesive strength can be regarded as a fundamental parameter of the coating. However, fatigue and wear considerably deteriorate the adhesive strength and shear stress of the coating and thus accelerate peeling. Hence, more attention should be paid to the fatigue strength and contact mechanics, rather than only the adhesive strength, in obtaining a long lifetime of the coating.

## Figures and Tables

**Table 1:** Deposition parameters of the microcrystalline diamond (MCD) coatings.

Item	MCD	
	Nucleation	Growth
Acetone/H_2_ flow (sccm)	80/200	90/200
Pressure (kPa)	1.5–2.0	4.0–5.0
Bias current (A)	4.0	3.0
Duration (h)	0.5	4
